# Hard then, harder now: internal medicine residents’ moral distress pre and amidst COVID-19

**DOI:** 10.1186/s12910-025-01274-6

**Published:** 2025-10-17

**Authors:** Harriet Fisher, Stephanie McLaughlin, Tavinder Ark, Sondra Zabar, Katharine Lawrence, Kathleen Hanley

**Affiliations:** 1 NYU Grossman School of Medicine, 550 1st Ave, New York, NY USA; 2 University of Washington, Seattle, USA; 3 Data Science Lab, Kern Institute, Medical College Wisconsin, Milwaukee, USA; 4 Department of Internal Medicine, Division of General Internal Medicine and Clinical Innovation, Director, Standardized Patient Program, New York, USA; 5 Department of Population Health, NYU Grossman School of Medicine, New York, USA; 6 Department of Medicine at NYU Grossman School of Medicine, Primary Care Internal Medicine Residency Program, New York, USA

**Keywords:** Ethical dilemmas, Moral distress, Moral injury, Resource allocation, Health care inequities, Residency training

## Abstract

**Background:**

Moral distress, which occurs when the ethically correct action cannot be taken because of internal or external constraints, is associated with depression, burnout, and the desire to leave the healthcare profession among healthcare workers. This study compares internal medicine (IM) residents’ experiences of moral distress while caring for patients with COVID-19 in the year prior to and during the first year of the COVID-19 pandemic.

**Methods:**

This is a mixed methods prospective observational cohort study that enrolled IM residents on a rolling basis beginning December 2018. Moral distress was evaluated via the validated Moral Distress Score-Revised (MDS-R) and Measure of Moral Distress for Healthcare Professionals (MDD-HP) and open-ended questions every 4-months via online surveys and through five resident focus groups. The moral distress scores (MDS) before and during the COVID-19 pandemic were compared using paired t-tests. Transcripts and free text were independently coded by investigators and analyzed by major themes and sub-themes.

**Results:**

Forty-five residents (42% response rate) completed at least one survey during the pre-COVID and COVID periods. Analysis revealed no significant difference in individuals’ pre-to-COVID mean MDS, but significant differences between the overall MDS mean intensity scores pre-to-COVID (62.2 to 68.3 *p* < .05). Of the 36 items, there was a significant mean increase in two items: (1) continue to participate in care for a hopelessly ill person who is being sustained on a ventilator, when no one will decide to withdraw life support (3.89 to 5.91, *p* < .05); fear of retribution if I speak up (2.13 to 4.20, *p* < .05). Qualitive findings included the exacerbation of existing moral distress and the emergence of new drivers of moral distress, including personal protective equipment, visitor policies, lack of moral framework, and tension between protecting one’s own health and caring for others.

**Conclusions:**

The results of this preliminary analysis suggest that the COVID-19 pandemic exacerbated pre-existing experiences of moral distress and brought to light new and different morally distressing situations for trainees. This analysis of the impact of the pandemic is valuable not only for identifying leverage points for intervention, but also for informing future crisis preparedness and cultivating moral resilience in trainees and the healthcare workforce.

**Supplementary Information:**

The online version contains supplementary material available at 10.1186/s12910-025-01274-6.

## Background

Moral distress is defined as the psychological distress that arises when clinicians recognize the ethically appropriate action but are unable to act due to internal or external constraints. First described in the nursing literature in the 1980 s, it is increasingly recognized as a pervasive phenomenon across healthcare disciplines: including physicians, nurses, paramedics, social workers, and home health staff [1–7]. The definition of moral distress has evolved over time, from its original focus on institutional barriers – including policies or hierarchies that prevent clinicians from acting in alignment with their moral or ethical beliefs – to include a broader range of systemic and situational factors that impede ethical action [8–10]. Our study emphasizes the traditional definitions and conceptualizations of moral distress, specifically Jameton’s 1984 definition (the psychological distress of being in a situation in which one is constrained from acting on what one knows to be right but explores its evolving meaning to frame the experiences of residents providing clinical care during the COVID-19 pandemic [11].

Research prior to the COVID-19 pandemic identified moral distress as a significant contributor to clinician burnout, turnover, and compromised patient care, particularly in high-stakes settings like intensive care and end-of-life care. Higher rates of moral distress are associated with emotional turmoil, depression, burnout, and the desire to leave the healthcare profession among nurses, physicians, and other healthcare workers [3, 12–14] The pandemic dramatically intensified these challenges, with studies documenting increased prevalence and severity of moral distress across healthcare settings and professions [15–17]. A survey of 488 intensive care unit (ICU) nurses between October 2020 and January 2021 revealed moderate-to-high moral distress, with higher levels associated with ventilator shortages, ICU experience, personal protective equipment (PPE) shortages, and lack of perceived support from administration [18]. In the fall of 2020, an ACP survey of almost 200 internal medicine physicians revealed generally low levels of moral distress, though 13% of respondents had very high levels, but the study could not characterize pre-COVID moral distress nor qualitative experience [19]. By 2021 a survey of over 300 Veterans Affairs (VA) internal medicine attendings, 81% reported moral distress during peak COVID-19 pandemic [20]. These studies, and others, are part of an important growing body of work to describe moral distress in healthcare workers, but to date no work has longitudinally characterized moral distress in residents. This is notable, given literature suggests that the rigid hagiarchies of training and residents’ real or perceived lack of clinical expertise make residents especially vulnerable to moral distress [9, 21]. 

In this study, qualitative and quantitative methods were employed to investigate the impact of the COVID-19 pandemic on moral distress among internal medicine residents who provided patient care both prior to and during the early stages of the pandemic. This study is unique in longitudinally characterizing moral distress among residents both before and during the pandemic, providing rare longitudinal insight into how acute system-level stressors amplify pre-existing vulnerabilities in this population.

## Methods

### Setting and sample

This study was conducted at a large urban public hospital. Eligible participants for this study included all post-graduate year (PGY) 1, 2, and 3 Internal Medicine residents who participated in the care of patients during the pandemic period of March-September 2020. During the acute pandemic period beginning in March 2020, the entire Internal Medicine house staff workforce was deployed to inpatient wards and ICU services to contribute to the direct care of over 5,000 patients hospitalized with the virus.

### Data collection

After completing an internal review board (IRB) approved written consent form through RedCap, residents completed a baseline moral distress survey and then follow-up surveys at 4-month intervals. Residents were also invited to participate in one of five focus groups. The baseline survey included residents’ gender, age, religions and training-related information. Surveys contained both open-ended questions related to the experience of moral distress as well as closed-ended questions about specific situations which may cause moral distress for residents; a validated instrument, the 21-item Measure of Moral Distress Scale-Revised (MDS-R), and five additional items from the Measure of Moral Distress in Healthcare Professionals (MMD-HP), a revision of the MDS-R, was used within the closed-ended question section to evaluate frequency and intensity of distress associated with specific situations experienced or witnessed [22]. The authors of this paper reached out to the author of this validated measure who explicitly consented to our use. The instrument was selected because at the time of study development it was the most used instrument in the literature, Surveys were distributed at four-month intervals in an effort to assess residents after completing a period of varied care (outpatient, wards, ICU). Moral distress scores consist of frequency score (0–4) multiplied by intensity score (0–4) with higher score indicating higher levels of moral distress.

Five focus group were conducted to explore residents’ definition of moral distress and experiences of self-identified morally distressing situations. The study purpose and consent procedure were included in the email invitation and again prior to the start of the focus group. The participants gave verbal consent for audio-recording prior to the focus group discussion. In each focus group, trained facilitators consented participants and then conducted semi-structured interviews in which they shared the literature definition of moral distressed and offered six prompts which focused on experiences with and consequences of moral distress. The interview questions were based on prior literature research. These questions included “What clinical situations or cases have caused you the most moral distress while on inpatient wards in residency? What clinical situations or cases have caused you the most moral distress while in the ICU during residency?”

### Quantitative data analysis

For the purpose of this analysis, surveys completed between March 15, 2019 and March 14, 2020 were defined as “pre-COVID.” “COVID” surveys were completed March 15, 2020 through March 14, 2021. Residents were included in analysis if they had completed at least one survey in both the pre-COVID and COVID periods. Quantitative analysis of survey results included descriptive statistical analysis, reporting mean and standard deviation for continuous variables and frequencies for categorical variables. Because surveys were administered every four months, and the enrollment was ongoing, participants completed different numbers of surveys (range, 1–4) during the pre and COVID periods. To account for this range, the mean value of MD-score survey responses for any individual who completed > one survey during the pre- or COVID period were used.

### Qualitative data analysis

Transcripts of audio recorded focus groups and free text from qualitative survey questions were independently coded by investigators (HF, KH, SM) through Dedoose^®^ software prior to meeting through a conventional content analysis-based approach [23]. Discrepant themes and codes were reviewed and iteratively revised by the full research team to establish clarity and consensus. Dedoose^®^ was used for data management.

### Mixed methods

Authors analyzed quantitative and qualitative data concurrently as part of a convergent mixed-methods design. The quantitative and qualitative analytic process is detailed above. The research team compared quantitative and qualitative themes in these mixed methods process to confirm alignment of results.

## Results

Of 108 eligible residents, 45 completed at least one survey during both the pre-COVID and COVID periods (42% response rate). The breakdown was as follows Male (51%), Female (49%); PGY1 29%, PGY2 34%, PGY3 27%, Postgraduate 5%; Not-specified 6%; Age range 25–35. The mean pre-COVID and COVID overall moral distress score, intensity score and frequency scores are reported in Table 1. There was no significant difference in moral distress scores or frequency of scores, but there was a significant increase in intensity of scores over this period. Table 2 reports the mean pre and COVID scores by individual item for which there was a significant mean increase in two items: (1) continue to participate in care for a hopelessly ill person who is being sustained on a ventilator, when no one will decide to withdraw life support (3.89 to 5.91, *p* <.05); (2) fear of retribution if I speak up (2.13 to 4.20, *p* <.05).

Focus group participants were equally distributed as follows across five focus groups: gender (six female, four male) and year (zero PGY1, five PGY2, eight PGY3). Qualitative analysis revealed two major themes: Pre-existing Drivers of Moral Distress and New Drivers of Moral Distress sub-themes. Within these two major themes ten sub themes were identified, which are detailed below.

**Table 1:** Change in composite (26 item) moral distress scores for 45 respondents

	Pre-COVID (*N* = 45)	COVID (*N* = 45)	Significance (paired t-test)
Mean Intensity (0-104)	62.2 (SD = 13.7)	68.3 (SD = 13.2)	0.001
Mean Frequency (0-104)	38.04 (SD = 9.8)	38.5 (SD = 10.4)	0.67
Mean Overall (0-416)	92.8 (SD = 35.7)	100.4 (SD = 36.8)	0.07

**Table 2:** Paired T-Tests of 26 mean moral distress survey items

	Mean Intensity (0–4)		Mean Frequency (0–4)		Mean Overall MD (frequency x intensity) (0–16)	
*N* = 45 ***= *p* <.05	Pre	COVID	Pre	COVID	Pre	COVID
Item 1: Provide less than optimal care due to pressures from administrators or insurers to reduce costs.	2.33 *** (SD = 0.97)	2.80 *** (SD = 1.09)	1.67 (SD = 0.90)	1.36 (SD = 1.05)	4.22 (SD = 3.41)	4.04 (SD = 3.66)
Item 2: Witness healthcare providers giving “false hope” to a patient or family.	2.32 (SD = 0.95)	2.47 (SD = 0.86)	1.73 (SD = 0.94)	1.58 (SD = 0.95)	4.25 (SD = 3.14)	4.03 (SD = 3.13)
Item 3: Follow the family’s wishes to continue life support even though I believe it is not in the best interest of the patient.	2.64 (SD = 0.98)	2.78 (SD = 0.89)	2.24 (SD = 0.82)	2.41 (SD = 1.02)	6.06 (SD = 3.26)	6.69 (SD = 3.69)
Item 4: Initiate extensive life-saving actions when I think they only prolong the dying process.	2.49 (SD = 0.99)	2.88 (SD = 0.89)	2.18 (SD = 0.75)	2.35 (SD = 1.00)	5.59 (SD = 3.19)	6.95 (SD = 3.99)
Item 5: Follow a family’s request not to discuss death with a dying patient who asks about dying.	2.22 (SD = 1.30)	2.51 (SD = 1.12)	0.56 (SD = 0.73)	0.43 (SD = 0.64)	1.16 (SD = 1.96)	1.04 (SD = 1.82)
Item 6: Carry out a supervising or more senior healthcare provider’s request for what I consider to be unnecessary tests and treatments.	1.76 (SD = 0.77)	2.11 (SD = 0.94)	2.00 *** (SD = 0.089)	1.59 *** (SD = 0.83)	3.58 (SD = 2.38)	3.37 (SD = 2.56)
Item 7: Continue to participate in care for a hopelessly ill person who is being sustained on a ventilator, when no one will make a decision to withdraw life support.	2.53 (SD = 1.10)	2.78 (SD = 0.96)	1.44 *** (SD = 0.93)	2.08 *** (SD = 1.09)	3.89 *** (SD = 2.99)	5.91*** (SD = 3.95)
Item 8: Avoid taking action when I learn that a healthcare provider colleague has made a medical error and does not report it.	2.28 *** (SD = 1.21)	2.76 *** (SD = 1.16)	0.48 (SD = 0.68)	0.42 (SD = 0.65)	0.99 (SD = 1.52)	0.99 (SD = 1.45)
Item 9: Assist a healthcare provider who in my opinion is providing incompetent care.	2.39 9 (SD = 1.21)	2.69 (SD = 1.12)	1.13 (SD = 0.97)	0.99 (SD = 0.82)	2.73 (SD = 3.05)	2.57 (SD = 2.44)
Item 10: Be required to care for patients I don’t feel qualified to care for.	2.42 (SD = 1.09)	2.72 (SD = 1.10)	1.53 (SD = 0.97)	1.53 (SD = 0.95)	4.05 (SD = 3.59)	4.45 (SD = 3.52)
Item 11: Witness medical trainees preform painful procedures on patients solely to increase their skill	2.19 (SD = 1.22)	2.58 (SD = 1.22)	0.91 (SD = 0.96)	0.59 (SD = 0.82)	1.53 (SD = 2.08)	1.30 (SD = 2.15)
Item 12: Provide care that does not relieve the patient’s suffering because another healthcare provider fears that increasing the dose of pain medication will cause death.	2.17 *** (SD = 1.01)	2.64 *** (SD = 1.00)	0.81 (SD = 0.88)	0.75 (SD = 0.87)	1.78 (SD = 2.25)	2.10 (SD = 2.67)
Item 13: Follow a supervising or more senior healthcare provider’s request not to discuss the prognosis with the patient or family.	2.45 (SD = 1.29)	2.74 (SD = 1.12)	0.38 (SD = 0.67)	0.25 (SD = 0.48)	0.95 (SD = 2.01)	0.59 (SD = 1.28)
Item 14: Increase the dose of sedatives/opiates for an unconscious patient that I believe could hasten the patient’s death.	1.77 (SD = 1.30)	2.11 (SD = 1.17)	0.72 (SD = 0.92)	0.74 (SD = 0.94)	0.82 (SD = 1.47)	1.32 (SD = 2.11)
Item 15: Take no action about an observed ethical issue because the involved staff member or someone in a position of authority requested that I do nothing.	2.62 *** (SD = 1.24)	3.17 *** (SD = 0.97)	0.37 (SD = 0.66)	0.31 (SD = 0.61)	0.97 (SD = 2.05)	1.00 (SD = 2.02)
Item 16: Follow the family’s wishes for the patient’s care when I do not agree with them, but do so because of fears of a lawsuit.	2.44 (SD = 1.07)	2.71 (SD = 0.96)	0.84 (SD = 0.98)	0.93 (SD = 0.91)	2.11 (SD = 2.71)	2.71 (SD = 3.02)
Item 17: Work with other healthcare providers who are not as competent as the patient care requires.	2.18 *** (SD = 1.11)	2.60 *** (SD = 0.90)	1.52 (SD = 0.99)	1.47 (SD = 0.80)	3.46 (SD = 3.52)	3.80 (SD = 2.73)
Item 18: Witness diminished patient care quality due to poor team communication.	2.37 (SD = 0.98)	2.66 (SD = 0.97)	1.84 (SD = 0.99)	1.83 (SD = 0.84)	4.57 (SD = 3.71)	5.02 (SD = 3.43)
Item 19: Ignore situations in which patients have not been given adequate information to ensure informed consent.	2.18 (SD = 1.01)	2.44 (SD = 1.02)	1.05 (SD = 0.99)	0.93 (SD = 0.96)	2.54 (SD = 2.91)	2.16 (SD = 2.35)
Item 20: Witness patient care suffer because of a lack of provider continuity.	1.99 (SD = 1.02)	2.32 (SD = 1.04)	2.19 (SD = 1.08)	2.26 (SD = 1.04)	4.68 (SD = 3.72)	5.71 (SD = 4.24)
Item 21: Work with levels of healthcare provider staffing that I consider unsafe.	2.66 (SD = 0.99)	2.97 (SD = 0.95)	2.28 (SD = 1.29)	2.20 (SD = 1.10)	6.62 (SD = 5.22)	6.77 (SD = 4.38)
Item 22: Be required to care for more patients than I can safely care for.	2.61 (SD = 0.95)	2.94 (SD = 1.02)	1.80 (SD = 1.02)	1.80 (SD = 1.00)	5.05 (SD = 4.01)	5.58 (SD = 4.02)
Item 23: Experience compromised patient care due to lack of resources/equipment/bed capacity.	2.66 *** (SD = 0.90)	3.02 *** (SD = 0.93)	2.56 (SD = 1.05)	2.18 (SD = 1.15)	7.24 (SD = 4.55)	6.80 (SD = 4.66)
Item 24: Be required to overemphasize tasks and productivity or quality measures at the expense of patient care.	2.32 *** (SD = 0.90)	2.57 *** (SD = 1.04)	1.87 (SD = 1.16)	1.54 (SD = 1.16)	5.03 (SD = 4.32)	4.46 (SD = 4.28)
Item 25: Experience lack of administrative support for a problem that is compromising patient care.	2.42 *** (SD = 1.08)	2.75 *** (SD = 1.06)	1.95 (SD = 1.21)	1.80 (SD = 1.14)	5.42 (SD = 4.92)	5.36 (SD = 4.38)
Item 26: Fear retribution if I speak up.	2.29 *** (SD = 1.16)	2.73 *** (SD = 1.21)	0.92 *** (SD = 0.98)	1.38 *** (SD = 1.31)	2.13 *** (SD = 2.75)	4.20 *** (SD = 2.82)

Select quotes that exemplify the major themes and subthemes identified in the survey free-text and focus groups can be found below and more quotes can be found in Appendix 1.

### Exacerbating pre-existing moral distress

Residents described many morally distressing realities that were present before the onset of the COVID-19 pandemic [24]. Discrepancies in performing medical codes (e.g. CPR) and end-of-life care, the exacerbation of inequality in care between public and private institutions, and inadequate resources were frequently described. While not felt to be new during the COVID-19 pandemic, these sources of moral distress were perceived to be exacerbated by the pandemic. As one resident wrote, “I felt like none of the sources of moral distress were actually new in a pandemic. They were just all worse and more obvious, and all of the social disparities, and how were more obvious.”

#### Discrepancies in who gets coded/end of life

Residents reported moral distress in response to the variation in end-of-life between those with without COVID, which led residents to feel that care was inconsistent and sometimes not aligned with patients’ values or best interests.

“I think codes were treated very differently during this time…like if it was a COVID (positive)patient coding versus a negative patient coding, and, like, the differences in how people responded and how fast they responded.”

#### Inequitable resource distribution

Residents who rotated in differently resourced hospitals reported moral distress in providing different levels of care based on resource availability, instead of clinical need.

“It’s really just like the social determinants of health of why this person ended up in the hospital in the first place… and all these other extenuating factors that obviously we all knew about before COVID, but especially was highlighted in this very extreme time, just how terribly unfair it is that the circumstances of your birth just so vastly dictate your health.”

#### Inadequate resources

Residents reported that inadequate hospital resources – including shortages of staff and ventilators – resulted in compromised care which residents reported as a driver of moral distress.

“Patients dying and their deaths could have been preventable if the hospital had basic resources.”

### New drivers of moral distress

Residents also described new morally distressing realities that emerged during the pandemic. These included distress around personal protective equipment, family visitation policies, a tension between protecting one’s own health and caring for others, loss of learning time, new manifestations of burnout, and revelations around institutional values. For instance, PPE policies were not captured in the moral distress survey, but comments from residents suggested that the lack of clear PPE policies and lack of moral clarity around managing their own health versus patient health drove moral distress in unprecedented ways.

#### Personal protective equipment (PPE) policy/lack of policy

Residents described how the lack of clear policy on what PPE was available and where to access it fostered interprofessional tension and eroded institutional trust.

“The [hospital] was like a free-for-all. You tried to find them (masks). It was like a scavenger hunt around the hospital…it created this rift between the doctors and nurses that was due to, again, poor institutional planning, because no one had told anyone where we were supposed to get our masks.”

“Again, that’s just unfortunate because we’re trying to do the best we can, all of us, and we’re all trying to be safe, and we just want to protect us and protect patients, and when there’s no clear instruction of what to do, it’s difficult.”

#### Visitor policy/lack of policy

Residents described how implementing the visitor policy, which required residents often choose just one family member to be with a loved one dying of COVID-19 drove their moral distress.

“The visitor policy…it wasn’t clearly right in my opinion. It’s not like it was clearly wrong either….”

“I felt like letting the family say goodbye and have several visits over which to come to terms with the patient’s poor prognosis would have been therapeutic for family members and honestly would have helped them realize that further aggressive care wouldn’t have helped.”

#### Insufficient moral framework

When reflecting on moral distress during this period residents remarked that they felt uncomfortable with the level or standard of care they had provided during the pandemic and wished they had had a clearer moral framework that allowed for variation in care, which could have alleviated this moral distress.

“I actually don’t know if there’s literature, if it’s acceptable to practice a different standard of care when there’s very extenuating circumstances like a pandemic. I actually think that would make me probably feel better, because I think I wonder if it was right.”

#### Conflict over concern for one’s well being

Residents reported feeling moral distress and guilt when they were concerned for their own health and well-being while caring for people sick with COVID-19.

“Should keep your head down and continue working ‘cause you’re a physician, and you’re held to a higher standard and now’s your chance. And there’s that, I guess that moral distress that comes in, it’s, like, well, so if I don’t agree with that, am I not a good person? Did I not do this for the right reasons?”

#### Loss of academic time/potential impact on training

Residents reflected on the loss of didactic and training time during the COVID-19 pandemic and the sense of moral distress and conflict they experienced over this reality.

“We were transitioning to being PGY2’s, which is kind of a big jump in responsibility and preparation and knowledge, and so having the absence of didactics and structured teaching and preparation, especially with that timeline in place, was, at least for me, distressing.”

#### Burnout

Residents reported moral distress driven by their sense that that the system would not care for them in future pandemic waves.

“Now that the initial wave has passed, my biggest cause of moral distress is for the extent of clinician burnout prior to future waves. I’m scared that moving forward we won’t get as much external assistance, and we’ll only have burned out house staff providing care.”

#### Institutional values

Residents described how the COVID-19 pandemic and their institution’s response revealed its values and, when in tension with those of residents, created new moral distress.“

“I think part of the experience of the pandemic is a reminder that you can’t separate the patient care we do from the corporate structure you are within… I think going forward, I’m going to be inquisitive and pay attention to what kind of environment I’m in, where the priorities are, where the goals are, what kind of decisions are being, you know, what the focus is on physicians and whether we’re incorporated in decision making.”

## Discussion

Research on moral distress in residents is important because it addresses a critical driver of burnout and compromised patient care. This study provides a unique and timely analysis of moral distress during the COVID-19 pandemic by comprehensively examining the prevalence and drivers of moral distress before and during the pandemic in the same study population. The integration of quantitative and qualitative data from the same cohort enables a multidimensional understanding of how pandemic-specific factors, in addition to organizational and structural constraints shaped the moral distress of residents. This analysis of the impact pandemic is valuable not only for identifying leverage points for intervention and support, but also for informing future crisis preparedness, ethics education, and the cultivation of moral resilience among trainees and the broader healthcare workforce.

In quantitative analysis, residents’ mean global moral distress scores (intensity multiplied by frequency) did not change significantly during the pandemic. Our results (92.8 to 100.4) are similar in magnitude to physicians’ moral distress in non-pandemic times in the literature, including a 2019 study of over 600 physicians across specialties that found a mean physician MD of 96.3 (Epstein, 2019). Although our residents’ global MDS did not change, their global intensity scores changed significantly pre-COVID to COVID-19 from 62.2 (SD = 13.7) to 68.3 (SD = 13.2). This change was driven by 9 specific items (Table 2). The two that were highest during COVID-19 were “take no action about an observed ethical issue because the involved staff member or someone in a position of authority requested that I do nothing” and “experience compromised patient care due to lack of resources/equipment/bed capacity.**”** Global frequency scores did not change significantly, likely because two of the 26 items significantly increased in frequency and one of 26 significantly decreased. Specifically, “continue to participate in care for a hopelessly ill person who is being sustained on a ventilator, when no one will make a decision to withdraw life support” and “fear retribution if I speak up” both increased, while one item, “carry out a supervising or more senior healthcare provider’s request for what I consider to be unnecessary tests and treatments” significantly decreased, likely reflecting the decrease in supervision at the onset of the pandemic.

These items reveal a complex landscape of moral distress: independently caring for critically ill patients with a new disease which had unclear evidence and best-practices likely drove resident’s moral distress, perhaps creating a culture in which it was harder to speak up or dissent; and yet, this very opportunity for independence, may have decreased the moral distress of carrying out a “senior[‘s]” “unnecessary” care that existed pre-COVID. While there is limited literature quantifying moral distress before and during the COVID-19 pandemic, and no studies have longitudinally evaluated resident physicians, one study from Spain assessed moral distress in nurses and intensive care unit attending physicians using the MMD-HP-SPA (a Spanish version of the MDD-HP) before and during the pandemic. This study found that moral distress scores increased significantly among nurses but not among physicians during this period. The study did not clarify whether the physician group included trainees; if not, this may support the hypothesis that residents’ experiences of moral distress are more similar to those of nurses, who are also subject to hierarchical constraints and limited autonomy. Additionally, the study identified the ten MMD-HP-SPA items with the highest scores, six of which overlapped with the highest-scoring items in this current study [25]. This close alignment between high-scoring items in this study and those in the Spanish study support the gerenalizbilty of our findings.

Qualitative analysis revealed that many new drivers of moral distress, including the visitor policy, PPE policy, institutional policies, conflict over concern for one’s well-being, impact on training, and inadequate resources. The visitation policy, in particular, emerged over and over again. Specifically, residents spoke about the “cruelty” of implementing a black-and-white visitation policy in which an 18-year-old child could visit but a 16-year-old could not, or a patient who reported anxiety could have family members stay but a patient who benefited from a family member translation could not. Residents described the way in which restricting visitation restricted family member’s understandings of their loved ones’ sickness. In turn, they suggested that this made family members more likely to elect more “aggressive” care. This distress is echoed in the moral distress scores through the significant increase in frequency of moral distress around “continuing to participate in care for a hopelessly ill person who is being sustained on a ventilator, when no one will make the decision to withdraw life support.” These themes are reflected in other qualitative assessments of moral distress during the pandemic, including a 2020 mixed-methods assessment of over 2000 primary care, dental and behavioral health workers which found the two most frequent drivers of moral distress to be “patients not being able to receive the best or needed care, and patients and staff risking infection” during peak pandemic [17]. In addition, a 2021 mixed methods study of 191 VA clinicians which identified five key themes: (1) patient visitation restrictions, (2) anticipatory actions, (3) clinical uncertainty related to Covid, (4) resource shortages, and (5) personal risk of contracting Covid, which align entirely with sub-themes identified in this paper [20]. 

The COVID-19 pandemic confronted residents with a spectrum of moral challenges, ambiguities, and dilemmas, that disrupted their professional roles, responsibilities, and identities. In many ways, residents seemed to desire more supervision and structure to guide the morally ambiguous decisions they needed to make around personal protective equipment, coding policies and the broad acceptability of practicing a different standard of care during a pandemic. At the same time, there were moments where residents resented a lack of agency in morally ambiguous territory. Namely, residents wrote over and over about the distress of enforcing an unforgiving family visitation policy. This tension, between alleviating moral distress though clear moral frameworks and protocols, and empowering healthcare providers with the agency to make their own decisions in morally murky areas is crucial to consider to support health care providers in processing this pandemic and addressing the next [19]. 

It is devastating that the most significant driver of moral distress during the pandemic was a “fear of retribution if I speak up.” Indeed, a study of moral distress during COVID-19 by the American College of Physicians found that higher perceived organizational support (respondent belief that their health organization valued them) was most strongly associated with lower moral distress (*p* <.001) [19]. Medical educators, administrators, and policymakers must create a culture of transparency in which learners and leadership can grapple with the realities of moral stress and distress. while this is not black and white, and exponentially harder to navigate during a pandemic, residency and educational leadership must transparently communicate decisions and protocols that feed and driver moral distress. Understanding these drivers of moral distress will likely require providing space for residents to vocalize the moral distress they experience. Without doing so our community cannot possibly train, deploy, and support residents in this and future catastrophes, be they pandemics, natural disasters, or unforeseeable crises.

### Limitations

This study was limited in that the data were only collected from one residency program and there was not sufficient power to look at individual drivers of moral distress including gender, age, ward/unit location, attending structure, and hours worked. In addition, given only residents who completed surveys in both the pre and COVID-19 periods were included, a significant number of respondents (63/108) were not analyzed in this data set, this impacts the generalizability of this work as it a subset of a convenience sample.

## Conclusion

The results of this analysis suggest that the COVID-19 pandemic exacerbated pre-existing experiences of moral distress, especially when caring for people at the end of life and when fearing retribution for speaking up, and brought to light new and different morally distressing situations for trainees. Even as the pandemic wanes, these morally distressing realities will likely continue to play out in old and new paradigms. This analysis of the impact of the pandemic is valuable not only for identifying leverage points for intervention, but also for informing future crisis preparedness and cultivating moral resilience in trainees and the healthcare workforce. More research is needed to understand how programs and institutions can support residents in regaining and maintaining a sense of moral agency and professional identity without fear of retribution even amid morally distressing events. The medical community must continue to grapple with these quandaries in order to co-create a more compassionate and resilient healthcare system, for ourselves and our patients.

## Supplementary Information

Supplementary Material 1.

## Data Availability

The datasets used and/or analyzed during the current study are available from the corresponding author on reasonable request.
