# Supplementary material for: Hard then, harder now: internal medicine residents’ moral distress pre and amidst COVID-19
Source: BMC Med Ethics. 2025 Oct 17;26:139. doi: 10.1186/s12910-025-01274-6 (PMC12533463; doi:10.1186/s12910-025-01274-6)
Supplement: Supplementary file 1 — Supplementary Material 1. [file 12910_2025_1274_MOESM1_ESM.docx]

| Pre-Existing Drivers of MD | Discrepancies in who gets coded/ end of life | “I think codes were treated very differently during this time…like if it was a COVID patient coding versus a negative patient coding, and, like, the differences in how people responded and how fast they responded”    “it did feel like there was no consistency to who we were choosing to code for how long we were choosing to code them… you didn’t have to a framework to be like, “This is when we stop,” or “This is how we’re comparing this patient to this patient,” then it felt very subjective and arbitrary in the moment." |
| --- | --- | --- |
|  | Inequitable Resource Distribution | [it] was especially distressing to go from [an Academic medical center] to [a public hospital] and see a system that was very resource limited versus resource avid...seeing how people got very different care, and feeling the feeling that we should be able to do an ABG on someone that’s critically ill.    “I felt like none of the sources of moral distress were actually new in a pandemic. They were just all worse and more obvious, and all of the social disparities, and how were more obvious.” |
|  | Inadequate Resources | “clinicians form non-medicine specialties some of whom haven't done clinical medicine in quite some time, taking care of COVID pts in an unsafe manner”    “patients dying and their deaths could have been preventable if the hospital had basic resources.” |
| New Drivers of MD | PPE Policy/Lack of Policy | “The PPE policies were definitely distressing. Each hospital was different. I was at the XX. I think it was, from my experience, the worst there because there was no policy. XX sort of figured it out and had a thing in place where you went to this window and you would get your masks, and you could get a certain amount, and you at least knew how much you could use and how to get it.”    “The XX was like a free-for-all. You tried to find them. It was like a scavenger hunt around the hospital…it created this rift between the doctors and nurses that was due to, again, poor institutional planning, because no one had told anyone where we were supposed to get our masks”    “gaslighting by the administration that there were adequate dialysis machines, resources, and medications to care for these patients when in reality patients died regularly due to lack of access while in the ICU - multiple patients dying due to no HD-compatible beds in a unit that was supposedly an ICU” |
|  | Visitor Policy | “one patient had five kids, and her 18-year-old could come, but not her 16-year-old, and we tried to get an exception … but we weren’t able to.”    “In medicine, we try to talk about quality of life just as much as we try to talk about length of life. But then, policy makers who made these policies for the hospital didn’t prioritize, like, the quality that the patients had during this time in terms of having family visit.”    “I felt like letting the family say goodbye and have several visits over which to come to terms with the patient’s poor prognosis would have been therapeutic for family members and honestly would have helped them realize that further aggressive care wouldn’t have helped” |
|  | Insufficient Moral Framework | “I actually don’t know if there’s literature, if it’s acceptable to practice a different standard of care when there’s very extenuating circumstances like a pandemic. I actually think that would make me probably feel better, because I think I wonder if it was right” |
|  | Conflict Over Concern For One’s Well Being | “should keep your head down and continue working ‘cause you’re a physician, and you’re held to a higher standard and now’s your chance. And there’s that, I guess that moral distress that comes in, it’s, like, well, so if I don’t agree with that, am I not a good person? Did I not do this for the right reasons? “ |
|  | Loss of Academic Time/Potential Impact on Training | “We were transitioning to being PGY2’s, which is kind of a big jump in responsibility and preparation and knowledge, and so having the absence of didactics and structured teaching and preparation, especially with that timeline in place, was, at least for me, distressing” |
|  | Burnout | “Now that the initial wave has passed, my biggest cause of moral distress is for the extent of clinician burnout prior to future waves. I'm scared that moving forward we won't get as much external assistance and we'll only have burned out house staff providing care.” |
|  | Institutional Values | “I think part of the experience of the pandemic is a reminder that can’t separate the patient care we do from the corporate structure you are within… I think going forward, I’m going to be inquisitive and pay attention to what kind of environment I’m in, where the priorities are, where the goals are, what kind of decisions are being, you know, what the focus is on physicians and whether we’re incorporated in decision making.” |
|  |  |  |

*Appendix 1*
